# Vitamin B6 Doses on United States Dietary Supplement Labels Relative to the Tolerable Upper Intake Level: A Census of the National Institutes of Health Dietary Supplement Label Database

**DOI:** 10.7759/cureus.118244

**Published:** 2026-10-20

**Authors:** Brian Staiger

**Affiliations:** 1 Pharmacy, University at Buffalo, State University of New York, Buffalo, USA

**Keywords:** dietary supplement label database, dietary supplements, pediatric supplements, peripheral neuropathy, pyridoxine, regulatory science, supplement labeling, supplement safety, tolerable upper intake level, vitamin b6

## Abstract

Background

High intakes of vitamin B6 can cause peripheral neuropathy. The United States (US) tolerable upper intake level (UL) for adults is 100 mg/day, and several other jurisdictions now apply far lower limits. No study has measured where US supplement labels sit against the US UL.

Methodology

This census retrieved every label flagged on the market in the National Institutes of Health Dietary Supplement Label Database (DSLD) on October 4, 2026 (plus eight labels found by an additional search spelling on October 13, 2026) that listed vitamin B6 under any spelling, merged the separate records that DSLD keeps when one product has a different package size, label image or alternate version into a single product, computed the maximum daily dose from each label's own directions, and compared it with the UL for adults or for the stated age band and with the lower thresholds of 12 and 50 mg/day used elsewhere. Every product at or above a US limit was verified against its raw record. Proportions carry Wilson 95% confidence intervals (CIs).

Results

Of 16951 labels, 15091 stated a vitamin B6 amount, forming 11913 products (11367 adult). The median maximum directed dose of adult products was 10 mg/day (interquartile range 2.2 to 25 mg/day). Overall, 575 of 11367 adult products (5.1%; 95% CI 4.7% to 5.5%) directed 100 mg/day or more, 167 (1.5%) more than 100 mg/day, and 10 at least 500 mg/day, with the highest being 1000 mg/day as the compound weight of a pyridoxine salt. Of these 575, 323 (56.2%) were not sold as vitamin B6 or B-complex products. In addition, 4399 adult products (38.7%) exceeded 12 mg/day and 1034 (9.1%) exceeded 50 mg/day. Label directions on 22 products gave a stated child or teen age a dose above the UL for that age, up to 250 mg/day for ages 4 and older (UL 40 mg/day). Verification excluded 12 inconsistent DSLD records and corrected 21 mis-encoded serving records.

Conclusions

One in 20 adult vitamin B6 supplements on the US market directs at least the UL daily, mostly in products not sold as vitamin B6, and single tablets reach five times the limit; nearly two in five exceed the European limit of 12 mg/day. The findings support a neuropathy warning on high-dose labels, a reassessment of the 1998 UL, and attention to vitamin B6 content when neuropathy is evaluated.

## Introduction

Vitamin B6 is one of the few vitamins whose excess supplementation has a well-documented adverse effect. Sensory peripheral neuropathy from high-dose pyridoxine was described in 1983 in adults taking two to six g/day [1], and a 1987 series associated symptoms with lower doses taken for longer periods [2]. More recent case reports describe neuropathic symptoms at doses near or below 25 mg/day [3], and laboratory work points to a paradoxical mechanism in which high concentrations of pyridoxine inhibit enzymes that depend on its own active form, pyridoxal 5'-phosphate [4].

The United States (US) tolerable upper intake level (UL), set by the Institute of Medicine in 1998, is 100 mg/day for adults from food and supplements combined, with 30, 40, 60 and 80 mg/day for ages one to three, four to eight, nine to 13 and 14 to 18 years; it has not been revised [5]. Other authorities have moved since. The European Food Safety Authority (EFSA) set an adult UL of 12 mg/day in 2023 with peripheral neuropathy as the critical effect [6]. The Netherlands limited the vitamin B6 content of supplements to 21 mg per daily dose from October 2018, after which reports of neuropathy to its pharmacovigilance centre fell [7]. After a rise in neuropathy reports, Australia's Therapeutic Goods Administration decided in November 2025 that oral products supplying more than 50 mg per recommended daily dose will become pharmacist-only medicines from June 2027, with products above 200 mg per day remaining prescription-only [8]. The United Kingdom's National Health Service (NHS) advises the public not to take more than 10 mg/day from supplements unless a doctor advises otherwise [9].

In the US, federal law has barred the Food and Drug Administration since 1976 from setting maximum potency limits for vitamins and minerals in supplements, except in products represented for use by children under 12 years, by pregnant or lactating women, or for specific diseases [10]. Supplement labels carry no required warning about neuropathy. The National Institutes of Health (NIH) Dietary Supplement Label Database (DSLD) holds a structured transcription of the Supplement Facts panel, the serving record and the directions of every label it has collected, together with a market-status flag [11]. DSLD has been used to measure multivitamin products for young children against the UL [12], and a published case describes vitamin B6 toxicity from a daily multivitamin [13], but no study has measured where the whole US market sits against the UL, product by product, using the dose that each label itself directs.

We therefore censused every on-market DSLD label that lists vitamin B6 to answer four questions: how much vitamin B6 the directions on US supplement labels deliver per day; how many adult products reach or exceed the US UL, and how that count depends on how the daily dose is defined; which kinds of products carry those doses; and how many products direct children or teenagers to doses above the UL for their age. As context, the same products were measured against the European, Dutch, Australian and British thresholds.

## Materials and methods

Study design and data source

This is a cross-sectional census of product labels, reported with reference to the Strengthening the Reporting of Observational Studies in Epidemiology (STROBE) guidance where it applies to a study without human participants [14]. The source is DSLD, maintained by the NIH Office of Dietary Supplements, accessed through its public application programming interface (version 9) [15]. DSLD records are US government public-domain data. The snapshot was taken on October 4, 2026; DSLD changes weekly, so every count below refers to that date, except the eight addendum labels described under Cohort, which were pulled on October 13, 2026.

Cohort

Every label whose DSLD market status was "on market" and whose ingredient filter matched any of nine spellings (Vitamin B6, Pyridoxine, Pyridoxal, Pyridoxamine, P-5-P, P5P, Pyridoxal 5'-Phosphate, B6 and B-6) was enumerated. The filter matches DSLD's curated ingredient-group name as well as the printed ingredient name, which is why the nine spellings returned nearly identical sets and why printed variants such as pyridoxine hydrochloride, pyridoxal-5-phosphate or P5P are reached through their group; the union of the spellings was taken, and each label was then judged on its own ingredient rows. The search index returns at most 10,000 hits per query; larger queries were read twice, sorted by entry date ascending and descending, and the unique identifiers were required to equal the reported hit count. The union held 16,943 labels, and the complete record of each was downloaded. In reply to peer review, the search was repeated on October 13, 2026 with 20 further spellings (every one is listed with its hit count in the deposited search log): 19 returned no on-market label outside the union that had entered DSLD before the snapshot date, and one, pyridoxine alpha-ketoglutarate, returned eight such labels (entered 2012 to 2020), because DSLD files that salt under its own ingredient group rather than a vitamin B6 group. Those labels were added to the census as a dated addendum, giving 16,951 labels in all, and the tenth spelling is part of the search for any future snapshot. A Secure Hash Algorithm 256-bit (SHA-256) digest of every record fixes the snapshot and is published with the data.

Unit of analysis

DSLD keeps a separate label record when the same product has a different package size, a different label image or an alternate version; this article calls these records twin labels. Labels that DSLD links as "package size difference, same product", "image difference, same product" or "alternate version" were merged into one product, represented by the label with the newest entry date; a reviewer correction on any twin label applied to the product. Flavour variants remained separate products. For 332 products, the twin labels disagreed on the dose by more than 1%, and the newest label was used; two sensitivity analyses bracket that choice by counting a product as at or above the UL if any of its labels was, and only if every one of its labels was.

Vitamin B6 amount and daily dose

A supplement facts row was treated as vitamin B6 when its DSLD ingredient group, or without a group, its name, named vitamin B6 or a pyridoxine, pyridoxal or pyridoxamine form. Four rules kept an amount from being counted twice, each enforced by a real record. First, rows nested under a vitamin B6 row are form breakdowns (as pyridoxal 5'-phosphate) and were not added; the parent row carried the amount, and its children were summed only when the parent stated none. Second, when a label declared vitamin B6 with a per cent daily value, other vitamin B6 compound rows without their own per cent daily value were treated as its sources rather than extra vitamin B6. Third, the same ingredient declared for two serving bases, such as 25 mg per scoop and 50 mg per two scoops, counted once. Fourth, vitamin B6 compounds inside other rows, such as a blend's pyridoxine alpha-ketoglutarate or a breakdown mis-nested under niacin, were used only when the label had no top-level vitamin B6 amount. Separately declared sources that each carried a per cent daily value were summed. An amount not disclosed, typically inside a proprietary blend, was recorded as undisclosed and never imputed.

The daily dose came from DSLD's serving record: the amount per stated basis, scaled to the units per serving, multiplied by the servings per day. The maximum daily dose used the largest serving size and the most servings per day the directions allow; the minimum used the smallest of each; a single serving was also recorded. A label with no servings-per-day field was counted as one serving a day and flagged. A scaling guard caught entry slips in which an amount already stated per serving had been recorded per 1 mL of a 355 mL can. A labelled amount that disagreed with the implied daily value base by more than a factor of three was flagged for review. Rows stating a pyridoxal 5'-phosphate or pyridoxine alpha-ketoglutarate compound weight rather than a vitamin B6 amount were also converted to vitamin B6 content (molar mass ratios 0.685 and 0.537) for a sensitivity analysis; the main analysis takes the label as printed.

Benchmarks

The primary benchmark was the US UL [5], which applies to total intake from food and supplements. Because US adults obtain about 1.5 mg/day (women) to 2 mg/day (men) of vitamin B6 from food [16], a product supplying 100 mg/day takes total intake over the adult UL on its own; "at or above the UL" therefore means that the product alone supplies 100 mg/day or more, and "above the UL" means more than 100 mg/day. Children's and teen products were compared with the UL for their age band. Context thresholds were the EFSA UL of 12 mg/day [6], the Dutch legal maximum of 21 mg per daily dose [7], the Australian scheduling thresholds of 50 and 200 mg per recommended daily dose [8] and the British advice ceiling of 10 mg/day [9].

Children's and teen products

A product was a children's or teen product only when its name said so (kids, children, child, toddler, infant, junior, teen, preteen, tween) or when every DSLD target group was a child group; DSLD's target-group tags alone were not trusted, because they also mark adult energy shots and prenatal products as "children 4 or more years of age" (460 adult products carry such a tag). Of the 546 products classed as children's or teen products, 387 were named as such and 159 had only child target groups. Where the name and the tags disagreed, the name won: 14 products with a children's name carried only adult tags, and 88 carried both. Prenatal products were adult products. The age band came from the name or the target groups (teen, 14 to 18 years; preteen or tween, 9 to 13; toddler or "children more than 1 but less than 4", 1 to 3; otherwise 4 to 8), and each product was compared with the UL for its band using the smallest daily dose its directions allow, a conservative choice. Because products sold to adults can also print children's doses, every label supplying at least 30 mg/day whose directions or label statements mentioned a child or teenager was read in full, and each age-specific dose above the UL for the youngest age in that group was recorded with the directions quoted. The 30 mg/day screen is the lowest UL of any age band, so a label whose maximum directed dose is below it cannot exceed any child's UL; the screen reads DSLD's encoded maximum, so a label whose serving record understated a printed child direction could escape it.

Verification

Every product at or above a US limit was verified against its raw DSLD record: the labelled amount had to agree with the label's own per cent daily value, and a maximum that depends on several servings had to match the printed directions. Products at one serving a day whose amount agreed with the per cent daily value and carried no parser flag were accepted by rule; every other product, and every children's record, was read. Two kinds of decisions were made, each recorded with its reason and published with the data: a record whose amount contradicted the label's own per cent daily value was excluded as internally inconsistent, and a record whose servings DSLD had encoded wrongly had its dose set to what the printed directions say. Where printed directions gave a range, the largest amount the words allow was taken; a direction to take more only as a health professional advises was not counted beyond the printed maximum. Nothing was changed silently. All decisions were made by one reviewer, the author, reading each record with the generative artificial intelligence tool described below; no second reviewer repeated the verification independently. Every decision is published with its reason in the review log so that any of them can be checked, and the census was also run with no exclusion and no correction to show what the review changed.

Product categories

Categories were assigned from the product name and the DSLD product type, in this order: prenatal; B-complex (names containing B complex, B-50, B-75, B-100, B-150, super B, B vitamins and similar); multivitamin and mineral (the DSLD product type); magnesium; single-ingredient vitamin B6 (names containing B6, B-6, pyridoxine, P5P or P-5-P); multivitamin by name; energy, sports and weight; sleep, mood, stress and brain; hair, skin and nails; women's health; heart and homocysteine; immune; and other. The categories are approximate and serve only to describe where the high-dose products sit.

Statistical analysis

Counts and proportions are reported with Wilson score 95% confidence intervals (CIs) [17]. Because every on-market record was analysed, the proportions are exact for the database on the snapshot date, and the intervals carry no sampling error for the database itself. They are reported because the database is a changing and incomplete representation of the US market, with products entering and leaving every week and not every marketed product listed, so a snapshot is one state of that market; the intervals describe the precision with which a snapshot of this size estimates the market it represents and allow comparison with a later snapshot, another database or a sample survey. No hypothesis tests were performed. Sensitivity analyses recomputed the share of adult products at or above 100 mg/day using one serving rather than the daily maximum; the smallest directed daily dose; labels instead of products; labels entered in 2020 or later and before 2020; exclusion of labels carrying any parser flag; exclusion of labels with no servings-per-day field; only products whose DSLD target groups name adults; pyridoxal 5'-phosphate and pyridoxine alpha-ketoglutarate weights converted to vitamin B6 content; and the two twin-label rules described above. All analyses used Python 3.11 (standard library only).

Reproducibility and data availability

The raw snapshot (16951 label records as retrieved, with the search log and the addendum), the analysis code, the product-level outputs, the list of every product at or above a US limit with its DSLD link, and the review log of every exclusion and correction with its reason are deposited at Zenodo under a CC BY 4.0 license [18]. The pipeline restores the snapshot offline, checks every record against the published digests, and reproduces every number in this article byte for byte.

Use of generative artificial intelligence

A generative artificial intelligence tool (Anthropic Claude) was used under the author's direction to review the analysis code and to read the raw label records during verification. The author reviewed the results and accepts full responsibility for the work.

Ethical details

The study used publicly available product-label data and contained no human participants or patient-level information. Institutional review board review was not applicable.

## Results

From labels to products

Of 16951 on-market labels naming vitamin B6, 1573 had no vitamin B6 ingredient row on their record (text-match noise), 275 listed vitamin B6 without a disclosed amount, and 12 were excluded after review as internally inconsistent, leaving 15091 labels with a vitamin B6 amount. Merging twin labels gave 11913 products: 11367 adult products, 541 children's or teen products with an age band, and five infant products, for which no UL exists (Table 1).

**Table 1:** From labels to products in the NIH Dietary Supplement Label Database snapshot of October 4, 2026. NIH: National Institutes of Health; UL: tolerable upper intake level.

Step	Count
Labels flagged on the market that the nine original search spellings returned on October 4, 2026	16943
Labels added by the tenth spelling (pyridoxine alpha-ketoglutarate) on October 13, 2026	8
Labels in the census	16951
Labels with no vitamin B6 ingredient row (text-match noise), excluded	1573
Labels listing vitamin B6 with no disclosed amount (proprietary blend), excluded	275
Labels excluded after manual review as internally inconsistent records	12
Labels with a vitamin B6 amount	15091
Products after merging twin labels (package size, label image or alternate version)	11913
Adult products	11367
Children's and teen products with an age band	541
Infant products (no UL exists for infants)	5

The nine original search spellings were largely redundant: the single spelling vitamin B6 found 15078 of the 15091 labels with a vitamin B6 amount (99.9%), and only three labels came from one original spelling alone. The vitamin B6 rows of these labels carried 51 distinct printed names under 19 DSLD ingredient groups; 17 rows had a printed name containing none of the nine spellings (for example, "PAK", "coenzymated" or "clorhidrato de piridoxina") and were reached through their group. The post-review probe of 20 further spellings added eight labels, which formed five products, four of them at or above the UL and the highest at 1,000 mg/day of pyridoxine alpha-ketoglutarate.

Distribution of maximum directed daily doses

Among the 11367 adult products, the median maximum directed daily dose was 10 mg (10th percentile 1.4 mg; interquartile range 2.2 to 25 mg; 90th percentile 50 mg; 99th percentile 150 mg; maximum 1000 mg). Doses cluster at round numbers: 1241 products (10.9%) are exactly 2 mg/day, 1127 (9.9%) are exactly 10 mg/day and 838 (7.4%) are exactly 50 mg/day (Figure 1).

**Figure 1:**
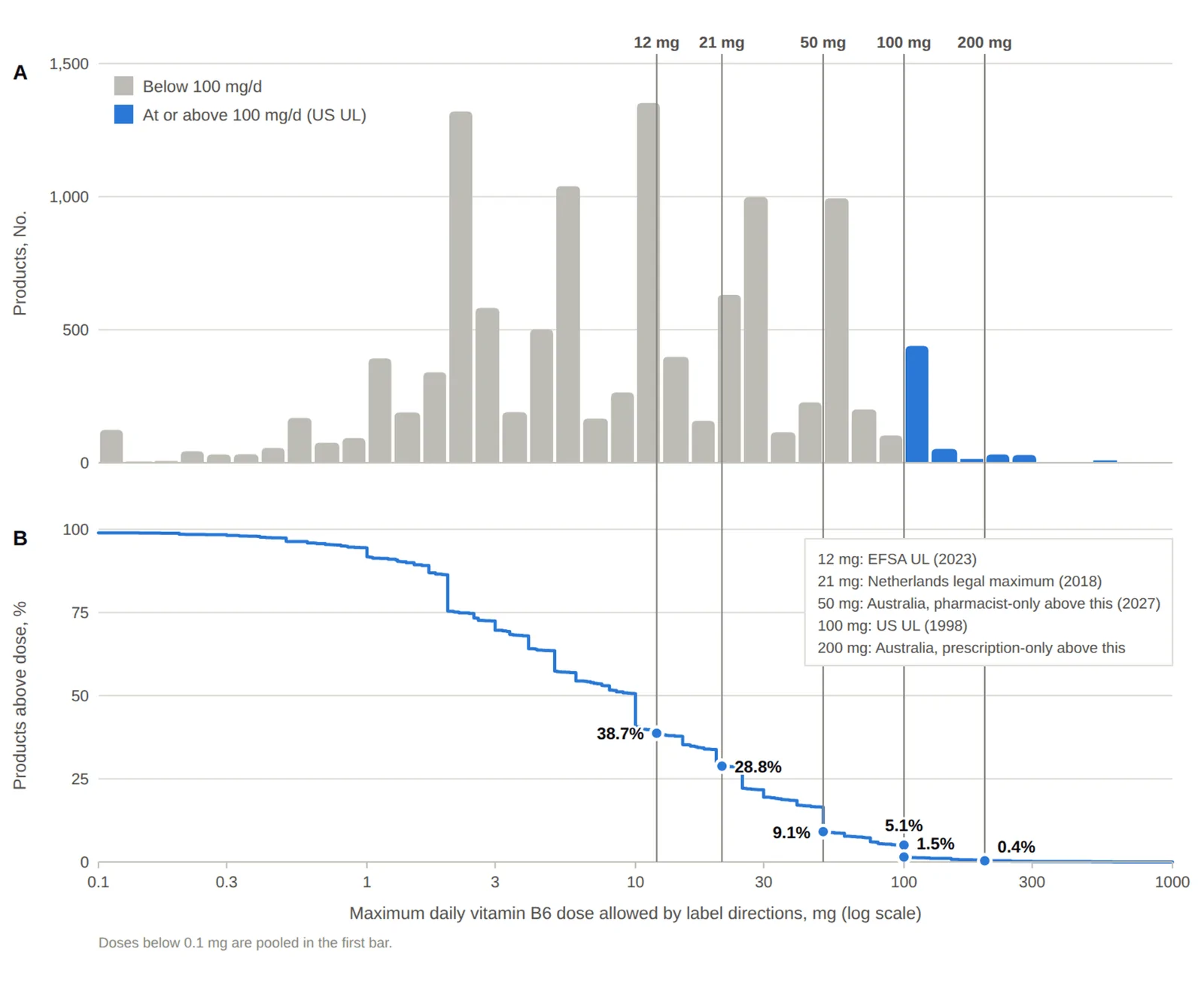
Maximum daily vitamin B6 dose allowed by label directions among 11,367 adult dietary supplement products. (A) Number of products per logarithmic dose interval (10 intervals per decade); the 92 products supplying less than 0.1 mg/day are pooled in the first interval, and the darker bars are at or above the US UL of 100 mg/day. (B) Percentage of products whose maximum directed dose exceeds each dose. Vertical lines mark the EFSA UL (12 mg/day) [6], the Dutch legal maximum (21 mg/day) [7], the Australian pharmacist-only threshold from June 2027 (50 mg/day) [8], the US UL (100 mg/day) [5] and the Australian prescription threshold (200 mg/day) [8]; the British advice ceiling of 10 mg/day [9] is not marked. In (B), the value at each line is the percentage of products above it; at 100 mg/day the upper value is the percentage at or above it and the lower value the percentage above it. EFSA: European Food Safety Authority; UL: tolerable upper intake level; US: United States.

Adult products relative to the US UL

In total, 575 of 11367 adult products (5.1%; 95% CI 4.7% to 5.5%) directed a maximum daily dose at or above the UL of 100 mg/day (Table 2). Of these, 408 (3.6% of adult products) directed exactly 100 mg/day, 300 of them in a single serving, and 167 (1.5%) directed more than 100 mg/day. A dose of at least 200 mg/day, twice the UL, was directed by 70 products (0.6%), and at least 500 mg/day by 10 (0.09%): 4 single-ingredient products supplying 500 mg of pyridoxine in one tablet taken once daily, two products whose directions reach 600 mg/day (a 200 mg tablet one to three times daily, and a multi-ingredient formula whose "alternative daily dosages" run to nine units), and 4 products that state their vitamin B6 as the compound weight of pyridoxine alpha-ketoglutarate, at 500 to 1000 mg/day of the compound (about 268 to 537 mg as pyridoxine), the highest doses in the census. The 575 products came from 239 brands, the largest contributing 40 products, and 491 (85.4%) were tablets or capsules.

**Table 2:** Maximum directed daily vitamin B6 dose of 11,367 adult dietary supplement products relative to the US tolerable upper intake level of 100 mg/day, with sensitivity analyses. The maximum directed daily dose is the vitamin B6 per serving multiplied by the largest serving and the most servings per day the label's directions allow. Percentages are of the 11367 adult products unless a row gives its own denominator. CIs are Wilson score intervals. CI: confidence interval; DSLD: Dietary Supplement Label Database; UL: tolerable upper intake level; US: United States.

Measure	Products, n (%)	95% CI, %
Maximum directed daily dose relative to the US UL of 100 mg/day		
At or above 100 mg/day	575 (5.1)	4.7 to 5.5
Exactly 100 mg/day	408 (3.6)	3.3 to 3.9
Above 100 mg/day	167 (1.5)	1.3 to 1.7
At or above 200 mg/day	70 (0.6)	0.5 to 0.8
At or above 500 mg/day	10 (0.09)	0.05 to 0.16
Sensitivity analyses, products at or above 100 mg/day		
Main analysis (maximum directed daily dose)	575 (5.1)	4.7 to 5.5
One serving only, not the daily maximum	403 (3.5)	3.2 to 3.9
Smallest daily dose the directions allow	467 (4.1)	3.8 to 4.5
Labels entered in 2020 or later (n = 7,190)	319 (4.4)	4.0 to 4.9
Labels entered before 2020 (n = 4,177)	256 (6.1)	5.4 to 6.9
Excluding labels with any parser flag (n = 10,951)	569 (5.2)	4.8 to 5.6
Excluding labels with no servings-per-day field (n = 11,041)	569 (5.2)	4.8 to 5.6
Only products whose DSLD target groups name adults (n = 11,365)	575 (5.1)	4.7 to 5.5
Pyridoxal 5'-phosphate and pyridoxine alpha-ketoglutarate compound weights converted to vitamin B6 content	575 (5.1)	4.7 to 5.5
Twin labels: any label of the product counted (n = 11,367)	591 (5.2)	4.8 to 5.6
Twin labels: every label of the product had to agree (n = 11,367)	568 (5.0)	4.6 to 5.4
Labels counted instead of products (n = 14,395)	806 (5.6)	5.2 to 6.0

The sensitivity analyses bracket the main estimate (Table 2). Counting one serving only gave 403 products (3.5%); the smallest directed daily dose gave 467 (4.1%); labels rather than products gave 806 of 14,395 (5.6%); labels entered in 2020 or later gave 319 of 7190 (4.4%) against 256 of 4177 (6.1%) for earlier entries; and converting pyridoxal 5'-phosphate and pyridoxine alpha-ketoglutarate compound weights to vitamin B6 content changed nothing at this threshold (575 products). Counting a product as at or above the UL if any of its twin labels was gave 591 (5.2%), and requiring every twin label to agree gave 568 (5.0%); 23 of the 310 adult products with disagreeing twin labels straddled the threshold.

Where the high doses sit

Single-ingredient vitamin B6 products and B-complex products carried the highest shares at or above the UL, 113 of 244 (46.3%) and 139 of 597 (23.3%), but together they held only 252 of the 575 products at or above the UL, 43.8% (Table 3). The other 323 (56.2%) were sold as something else: 51 multivitamin and mineral products (2.0% of 2,549), 13 women's health products (5.6% of 234), 29 sleep, mood, stress and brain products (4.1% of 703), and 200 products in no named category (4.3% of 4,675). Above the European threshold of 12 mg/day, the pattern was stronger: 3,794 of the 4,399 adult products above 12 mg/day (86.2%) were not vitamin B6 or B-complex products.

**Table 3:** Adult products at or above the US tolerable upper intake level and above the European threshold, by product category. Categories were assigned from the product name and the DSLD product type and are approximate. DSLD: Dietary Supplement Label Database; UL: tolerable upper intake level; US: United States; ZMA: zinc, magnesium aspartate.

Product category	Adult products, n	At or above the US UL of 100 mg/day [5], n (% of category)	Above the European UL of 12 mg/day [6], n (% of category)	Median maximum directed dose, mg/day
B-complex	597	139 (23.3)	402 (67.3)	50
Single-ingredient vitamin B6	244	113 (46.3)	203 (83.2)	55
Multivitamin and mineral	2,549	51 (2.0)	815 (32.0)	5
Sleep, mood, stress, brain	703	29 (4.1)	255 (36.3)	10
Women's health	234	13 (5.6)	134 (57.3)	20
Energy, sports, weight	1,357	12 (0.9)	374 (27.6)	5
Heart, homocysteine	196	7 (3.6)	111 (56.6)	15
Hair, skin, nails	270	7 (2.6)	59 (21.9)	5
Immune	132	3 (2.3)	43 (32.6)	10
Prenatal	327	1 (0.3)	74 (22.6)	4
Magnesium (including ZMA)	83	0 (0.0)	21 (25.3)	10
Other (no named category)	4,675	200 (4.3)	1,908 (40.8)	10
All adult products	11,367	575 (5.1)	4,399 (38.7)	10

Other jurisdictions' thresholds

Measured against the thresholds other authorities use, 4624 adult products (40.7%) exceeded the British advice ceiling of 10 mg/day, 4399 (38.7%) exceeded the EFSA UL of 12 mg/day, 3,277 (28.8%) exceeded the Dutch legal maximum of 21 mg/day, 1,034 (9.1%) exceeded the 50 mg/day above which Australia will require a pharmacist, and 45 (0.4%) exceeded the 200 mg/day above which Australia requires a prescription (Table 4).

**Table 4:** Adult products above the thresholds used by other jurisdictions. CI: confidence interval; EFSA: European Food Safety Authority; NHS: National Health Service.

Threshold	Jurisdiction and rule	Adult products above it, n (%)	95% CI, %
Above 10 mg/day	United Kingdom: NHS advice not to exceed this from supplements without medical advice [9]	4,624 (40.7)	39.8 to 41.6
Above 12 mg/day	European Union: EFSA tolerable upper intake level (2023) [6]	4,399 (38.7)	37.8 to 39.6
Above 21 mg/day	Netherlands: legal maximum in supplements (2018) [7]	3,277 (28.8)	28.0 to 29.7
Above 50 mg/day	Australia: pharmacist-only above this per recommended daily dose from June 2027 [8]	1,034 (9.1)	8.6 to 9.6
Above 200 mg/day	Australia: prescription-only above this per recommended daily dose [8]	45 (0.4)	0.3 to 0.5

Children and teenagers

DSLD held 541 children's or teen products with an age band: 325 for ages one to three, 158 for ages four to eight, two for ages nine to 13 and 56 for ages 14 to 18. At the smallest dose their directions allow, four exceeded the UL for their age band (seven at the largest). Reading every label supplying 30 mg/day or more whose directions or statements named a child or teen age identified 22 products, including those four, that direct a stated child or teen age to a dose above the UL for that age, 28 age-specific directions in all (Table 5). The highest was 250 mg/day for ages four and older, 6.2 times the 40 mg/day UL for ages four to eight. Of these 22 products, 10 from one manufacturer state that they are "formulated for adults and children four years and older" and give everyone a single dose of 50 to 250 mg/day. Other examples include a weight-gain syrup and matching capsules directing ages 2 to 12 to 30 to 45 mg/day and ages 12 to 18 to 90 mg/day, a chewable directing ages 4 to 11 to 60 mg/day, and a product for female teenagers directing 90 mg/day against a UL of 80 mg/day for ages 14 to 18.

**Table 5:** Doses that label directions give to a stated child or teen age that are above the US tolerable upper intake level for that age, each verified against the label record. Each row is one age-specific direction; a label with two age groups appears twice. Products are identified by their DSLD label number, which opens the full record at dsld.od.nih.gov/label/{number}. DSLD: Dietary Supplement Label Database; UL: tolerable upper intake level; US: United States.

DSLD label	Age stated on the label	Daily dose for that age, mg	US UL for the youngest age in the group, mg	Directions as printed (dose computed by the reviewer in parentheses)
297255	adults and children 4 and older (one dose for all)	250	40 (ages 4 to 8)	Take two full scoops (2.5g) daily or as directed by a physician. Caution: Formulated for adults and children four years and older. For children under four, consult a physician.
317770	adults and children 4 and older (one dose for all)	250	40 (ages 4 to 8)	Take three tablets per day or as directed by a physician. Caution: Formulated for adults and children four years and older. For children under four, consult a physician.
317769	adults and children 4 and older (one dose for all)	200	40 (ages 4 to 8)	One teaspoon (5 mL) daily or as directed by a physician. Caution: Formulated for adults and children four years and older. For children under four, consult a physician.
288173	over 12	132	60 (ages 9 to 13)	Over 12 years old: 2 capsules, 3 to 4 times daily (99 to 132 mg).
317773	adults and children 4 and older (one dose for all)	75	40 (ages 4 to 8)	Take two full scoops (2.5g) daily or as directed by a physician. Caution: Formulated for adults and children four years and older. For children under four, consult a physician.
333058	adults and children 4 and older (one dose for all)	75	40 (ages 4 to 8)	Take three tablets per day or as directed by a physician. Caution: Formulated for adults and children four years and older. For children under four, consult a physician.
8128	ages 4 and older	75	40 (ages 4 to 8)	Children ages 4 and older: 2 capfuls, morning and afternoon (75 mg).
177113	children 12 and older	102	60 (ages 9 to 13)	Adults and children 12 years and older take 2 capsules, three times daily (102 mg).
288173	ages 6 to 12	66	40 (ages 4 to 8)	6-12 years old: 2 capsules, twice daily (66 mg).
235656	children over 12	90	60 (ages 9 to 13)	Adults and children over the age of 12 take 3 capsules up to three times daily as needed (up to 90 mg).
247793	6 and older	60	40 (ages 4 to 8)	For individuals 6 or older, increase to 1 scoop 3 times per day (60 mg).
303950	ages 4 to 11	60	40 (ages 4 to 8)	4-11 years old: 1 tablet, twice daily (60 mg).
303950	over 12	90	60 (ages 9 to 13)	Over 12 years old: 1 tablet, 3 times daily (90 mg).
313013	ages 2 to 12	45	30 (ages 1 to 3)	(Age 2-12) 1 capsule, 2 to 3 times daily before meal (30 to 45 mg).
313013	ages 12 to 18	90	60 (ages 9 to 13)	(Age 12-18) 2 capsules, 3 times daily before meal (90 mg).
313014	ages 2 to 12	45	30 (ages 1 to 3)	(Age 2-12) 5ml, 2 to 3 times daily before meal (30 to 45 mg).
313014	ages 12 to 18	90	60 (ages 9 to 13)	(Age 12-18) 10ml, 3 times daily before meal (90 mg).
247793	children younger than 6	40	30 (ages 1 to 3)	For children younger than 6, increase to 1 or 2 scoops per day (20 to 40 mg).
261930	children 4 and up	50	40 (ages 4 to 8)	For children aged 4 and up, take 1 softgel 3 times per day (50 mg).
297238	adults and children 4 and older (one dose for all)	50	40 (ages 4 to 8)	Take two capsules daily or as directed by a physician. Caution: Formulated for adults and children four years and older. For children under four, consult a physician.
317701	adults and children 4 and older (one dose for all)	50	40 (ages 4 to 8)	One tablet daily or as directed by a physician. Caution: Formulated for adults and children four years and older. For children under four, consult a physician.
317763	adults and children 4 and older (one dose for all)	50	40 (ages 4 to 8)	Take two capsules daily or as directed by a physician. Caution: Formulated for adults and children four years and older. For children under four, consult a physician.
317764	adults and children 4 and older (one dose for all)	50	40 (ages 4 to 8)	One capsule daily or as directed by a physician. Caution: Formulated for adults and children four years and older. For children under four, consult a physician.
317776	adults and children 4 and older (one dose for all)	50	40 (ages 4 to 8)	Take one capsule daily or as directed by a physician. Caution: Formulated for adults and children four years and older. For children under four, consult a physician.
322048	children 4 and older	50	40 (ages 4 to 8)	Adults and children 4 years of age and older: one tablespoonful (15 mL) (50 mg).
8128	ages 2 to 3	37.5	30 (ages 1 to 3)	Children ages 2-3: 1 capful, morning and afternoon (37.5 mg).
23739	teens	90	80 (ages 14 to 18)	Take 4 tablets daily (90 mg).
33854	girls over 12	66.7	60 (ages 9 to 13)	As a dietary supplement for females above age 12: 1-2 capsules daily complement a balanced diet and lifestyle; 3-4 capsules daily provide a well-balanced maintenance and therapeutic level of supplementation (up to 67 mg).

Quality of the DSLD records

Verification covered 579 products at or above a US limit, and 303 label records were read in full. It excluded 12 records whose amount contradicted the label's own per cent daily value, mostly decimal slips (for example, 100 mg beside 500% of the Daily Value, which implies about 10 mg) and one "5.3 g" of vitamin B6 in a 26 g scoop, and it corrected 21 records whose servings DSLD had encoded wrongly, 17 downward and 4 upward. The dominant pattern was "two capsules twice daily" stored as a four-capsule serving taken twice, which doubles the dose. Without the review, the raw encoding would have given 597 adult products at or above the UL (5.2% of 11377), 184 above it, 84 at or above 200 mg/day and a maximum of 5300 mg/day; every one of the 12 exclusions and 13 of the corrections removed a product from the group at or above the UL, 1 correction moved a product into it, and the review therefore lowered the estimate from 597 to 575. The share of adult products at or above the UL was 6.1% for labels that entered DSLD before 2020 and 4.4% for those entered in 2020 or later, and the highest shares were among the earliest entries (9.7% in 2011 and 9.5% in 2012); the last three tables list the products at or above 200 mg/day (Table 6), every review decision (Table 7) and the figures by entry year (Table 8).

**Table 6:** Adult products whose label directions allow at least 200 mg/day of vitamin B6, twice the US tolerable upper intake level (70 products). The full list of the 575 products at or above 100 mg/day is in the deposited dataset. Products are identified by their DSLD label number, which opens the full record at dsld.od.nih.gov/label/DSLD (label number). The serving column shows the DSLD serving record as encoded; where a reviewer corrected it, the corrected dose is shown, and the decision appears in Table 7. DSLD: Dietary Supplement Label Database.

DSLD label	Maximum directed daily dose, mg	Vitamin B6 per serving, mg	Serving size and servings per day as encoded	Category
240740	1,000	500	3 capsules, 1 per day	Other
18772	600	200	3 tablets, 1 to 3 per day	Other
19485	600	300	3 capsules, 2 per day	Other
259150	600	200	2 capsules, 3 per day	Other
270870	600	200	1 tablet, 1 to 3 per day	B6 single-ingredient
8688	500	500	1 capsule, 1 per day	Other
272733	500	500	1 tablet, 1 per day	B6 single-ingredient
283092	500	500	1 tablet, 1 per day	B6 single-ingredient
303205	500	500	1 tablet, 1 per day	B6 single-ingredient
316292	500	500	1 tablet, 1 per day	B6 single-ingredient
8660	300	300	6 tablets, 1 per day	Women's health
9655	300	300	1 tablet, 1 per day	B6 single-ingredient
19607	300	300	6 tablets, 1 per day	Multivitamin
21810	300	100	2 to 6 tablets, 1 per day	Women's health
22149	300	100	2 to 6 tablets, 1 per day	Women's health
22200	300	100	2 to 6 tablets, 1 per day	Other
23708	300	300	1 tablet, 1 per day	B6 single-ingredient
72657	300	100	2 tablets, 3 per day	Other
231756	300	150	2 to 4 capsules, 1 per day	Other
304217	300	150	3 capsules, 2 per day	Other
208367	275	275	1 capsule, 1 per day	B6 single-ingredient
234813	275	275	1 capsule, 1 per day	B6 single-ingredient
7948	250	50	1 tablet, 1 to 5 per day	B6 single-ingredient
25387	250	250	3 taplets, 1 per day	Other
25510	250	250	1 tsp, 1 per day	Multivitamin
30583	250	250	1 capsule, 1 per day	B6 single-ingredient
51497	250	250	1 vegetable capsule, 1 per day	B6 single-ingredient
201657	250	250	1 vegetable capsule, 1 per day	B6 single-ingredient
220227	250	250	1 tablet, 1 per day	B6 single-ingredient
223437	250	250	1 capsule, 1 per day	B6 single-ingredient
247292	250	250	1 capsule, 1 per day	B6 single-ingredient
272037	250	250	1 tablet, 1 per day	B6 single-ingredient
297255	250	250	2.5 gram, 1 per day	Multivitamin
304945	250	250	1 vegetarian capsule, 1 per day	B-complex
317770	250	250	3 tablets, 1 per day	Multivitamin
319030	250	125	4 tablets, 2 per day	Multivitamin
328793	250	250	1 vegetarian capsule, 1 per day	B6 single-ingredient
333526	250	250	1 tablet, 1 per day	B6 single-ingredient
337268	250	250	1 capsule, 1 per day	B6 single-ingredient
215859	230	230	1 capsule, 1 per day	B6 single-ingredient
25759	225	225	5 ml, 1 per day	Multivitamin
258741	225	225	10 capsules, 1 per day	Other
11756	220	220	1 capsule, 1 per day	Other
258727	206	52	1 to 2 capsules, 2 per day	B-complex
304277	206	52	1 to 2 capsules, 2 per day	Other
6923	200	200	3 tablets, 1 per day	Women's health
8737	200	200	1 tablet, 1 per day	B6 single-ingredient
18461	200	100	5.3 grams, 1 to 2 per day	Energy, sports, weight
26398	200	100	4 tablets, 2 per day	Multivitamin
33952	200	50	1 to 2 capsules, 1 to 2 per day	B6 single-ingredient
36216	200	100	1 tablet, 2 per day	B6 single-ingredient
71984	200	50	1 to 4 capsules, 1 per day	Other
78506	200	100	1 tablet, 2 per day	B-complex
79462	200	200	1 vegetarian capsule, 1 per day	B6 single-ingredient
214500	200	200	1 tablet, 1 per day	B6 single-ingredient
240850	200	200	2 capsules, 1 per day	Other
243042	200	200	1 tablet, 1 per day	B6 single-ingredient
271690	200	50	2 to 4 capsules, 1 to 2 per day	Other
273854	200	200	1 tablet, 1 per day	B6 single-ingredient
292245	200	200	1 tablet, 1 per day	B6 single-ingredient
293897	200	200	1 capsule, 1 per day	B6 single-ingredient
295497	200	200	1 capsule, 1 per day	B6 single-ingredient
299293	200	100	2 capsules, 2 per day	Other
305154	200	50	1 to 2 capsules, 1 to 2 per day	B6 single-ingredient
305189	200	100	1 vegetarian capsule, 1 to 2 per day	B6 single-ingredient
306549	200	100	4 capsules, 2 per day	Other
317769	200	200	5 ml, 1 per day	Multivitamin
322299	200	100	5 capsules, 2 per day	Other
322598	200	200	12 pills, 1 per day	Other
332775	200	200	1 capsule, 1 per day	B6 single-ingredient

**Table 7:** Manual review decisions: records excluded as internally inconsistent and records whose dose was set to the printed directions, each with its reason. DSLD: Dietary Supplement Label Database; DV: Daily Value.

DSLD label	Decision	Reason
1179	Excluded	B6 entered as 5.3 g per 26 g scoop with no %DV; implausible as a vitamin B6 amount and unverifiable from the record (probably 5.3 mg).
2883	Excluded	38 mg B6 per packet contradicts the label's own 3% Daily Value (about 0.05 mg); data-entry error.
6220	Dose set to 50 mg/day	Label: take 2 packs daily (50 mg). DSLD encoded a 2-pack serving taken twice (100 mg).
23203	Dose set to 50 mg/day	Label: four capsules daily, two twice a day (50 mg). DSLD encoded a 4-capsule serving taken twice (100 mg).
26749	Dose set to 33.3 mg/day	Label (Spanish): 2 tablets 2 times a day (4 a day = 33 mg). DSLD encoded a 6-tablet serving taken twice (100 mg).
28977	Dose set to 50 mg/day	Label: one tablet 3 times daily (3 a day = 50 mg). DSLD encoded a 3-tablet serving taken 3 times (150 mg).
32903	Excluded	367 mg B6 per packet contradicts the label's own 33% Daily Value (about 0.6 mg); data-entry error.
33065	Dose set to 60 mg/day	Label: two capsules twice daily (4 a day = 60 mg). DSLD encoded a 4-capsule serving taken twice (120 mg).
33306	Dose set to 70 mg/day	Label: 2 capsules 2 times daily (4 a day = 70 mg). DSLD encoded a 4-tablet serving taken twice (140 mg).
33854	Dose set to 66.7 mg/day	Label: 1 to 4 capsules daily (up to 67 mg). DSLD encoded up to four 3-capsule servings (200 mg).
38648	Dose set to 50 mg/day	Label: 1 scoop (31 g) twice daily on days 1 to 4 (50 mg); the 3-times-daily step is a different powder. DSLD encoded up to 3 double scoops (150 mg).
59191	Dose set to 100 mg/day	Label: two capsules twice daily (4 a day = 100 mg). DSLD encoded a 4-capsule serving taken twice (200 mg).
74971	Dose set to 132 mg/day	Label: over 12 years old, 2 capsules 3 to 4 times daily (99 to 132 mg). DSLD encoded one 2-capsule serving a day (33 mg), the dose for ages 4 to 6.
75377	Dose set to 100 mg/day	Label: 1 to 4 tablets daily (up to 100 mg). DSLD encoded up to four 4-tablet servings (400 mg).
79861	Dose set to 100 mg/day	Label: two to four tablets daily (up to 100 mg). DSLD encoded up to four 4-tablet servings (400 mg).
183028	Dose set to 100 mg/day	Label: 1 caplet twice a day (2 a day = 100 mg). DSLD encoded a 2-caplet serving taken twice (200 mg).
228239	Excluded	50 mg B6 per scoop contradicts the label's own 5882% Daily Value (about 100 mg); the record is internally inconsistent.
233000	Dose set to 50 mg/day	Label: adults take 1 pack daily (50 mg). DSLD encoded up to two packs a day (100 mg).
240726	Dose set to 1000 mg/day	Addendum label (2026-10-13). DSLD encodes 3 capsules once a day (500 mg pyridoxine alpha-ketoglutarate per serving); the printed directions say three capsules upon rising and three before bed, so the maximum directed dose is 1,000 mg/day of the compound (about 537 mg as pyridoxine); the product's 2013 twin labels encode the same two servings.
240740	Dose set to 1000 mg/day	Addendum label (2026-10-13). DSLD encodes 3 capsules once a day (500 mg pyridoxine alpha-ketoglutarate per serving); the printed directions say three capsules upon rising and three before bed, so the maximum directed dose is 1,000 mg/day of the compound (about 537 mg as pyridoxine); the product's 2013 twin labels encode the same two servings.
241396	Dose set to 50 mg/day	Label: one tablet and one softgel daily (50 mg). DSLD encoded up to two tablets a day (100 mg).
247918	Excluded	75 mg B6 per teaspoon contradicts the label's own 375% Daily Value (about 7.5 mg); probable decimal slip.
259070	Excluded	150 mg B6 per 15 mL contradicts the label's own 2500% Daily Value (about 43 to 50 mg); data-entry error.
261837	Excluded	85 mg B6 per scoop contradicts the label's own 400% Daily Value (about 8 mg); probable decimal slip.
261838	Excluded	85 mg B6 per scoop contradicts the label's own 400% Daily Value (about 8 mg); probable decimal slip.
261839	Excluded	100 mg B6 per scoop contradicts the label's own 500% Daily Value (about 10 mg); probable decimal slip.
261840	Excluded	85 mg B6 per scoop contradicts the label's own 400% Daily Value (about 8 mg); probable decimal slip.
261841	Excluded	100 mg B6 per scoop contradicts the label's own 500% Daily Value (about 10 mg); probable decimal slip.
261842	Excluded	85 mg B6 per scoop contradicts the label's own 400% Daily Value (about 8 mg); probable decimal slip.
264221	Dose set to 45 mg/day	Label: usual adult dose 2 to 3 tablets at bedtime (30 to 45 mg). DSLD encoded up to three 3-tablet servings (135 mg).
266474	Dose set to 50 mg/day	Label: take 2 capsules per day (50 mg). DSLD encoded up to two 2-capsule servings (100 mg).
270868	Dose set to 150 mg/day	Label: one tablet one to three times daily (up to 150 mg). DSLD encoded up to two a day (100 mg).
332821	Dose set to 50 mg/day	Label: take 1 to 3 capsules daily (up to 50 mg). DSLD encoded up to 6 capsules (100 mg).

**Table 8:** Adult products by the year their label entered DSLD. The entry year is when DSLD recorded the label, not when the product was launched. DSLD: Dietary Supplement Label Database.

Year the label entered DSLD	Adult products, n	At or above 100 mg/day, n (%)	Above 12 mg/day, n (%)	Median maximum directed dose, mg/day
2011	145	14 (9.7)	69 (47.6)	10
2012	599	57 (9.5)	289 (48.2)	10
2013	655	41 (6.3)	301 (46.0)	10
2014	437	16 (3.7)	178 (40.7)	10
2015	377	20 (5.3)	150 (39.8)	10
2016	441	19 (4.3)	168 (38.1)	8
2017	525	41 (7.8)	232 (44.2)	10
2018	428	14 (3.3)	145 (33.9)	5
2019	570	34 (6.0)	191 (33.5)	6
2020	980	51 (5.2)	416 (42.4)	10
2021	1,083	41 (3.8)	389 (35.9)	10
2022	1,331	67 (5.0)	482 (36.2)	8
2023	1,151	56 (4.9)	429 (37.3)	8.5
2024	1,354	51 (3.8)	523 (38.6)	10
2025	1,291	53 (4.1)	437 (33.8)	6

## Discussion

Principal findings

In a complete census of the US supplement market as DSLD records it, one in 20 adult vitamin B6 products directed a daily dose at or above the US UL, one in 68 exceeded it from the product alone, and single tablets reached five times it. The estimate is robust to how the daily dose is defined: between 3.5% (one serving) and 5.6% (labels rather than products) of the market sits at or above the UL under every definition of dose or unit tried. Because food already supplies about 1.5 to 2 mg/day [16], a product at exactly 100 mg/day, the most common high dose, takes total intake over the UL by itself. More than half of the products at or above the UL were not sold as vitamin B6 or B-complex products, and more than four in five of the products above the European threshold were not, so consumers may take these doses without knowing it and clinicians evaluating neuropathy may not ask about them, a pattern already described in a case of vitamin B6 toxicity from a daily multivitamin [13].

Comparison with other jurisdictions and prior work

The doses other regulators have restricted are common on US labels. Nearly two in five adult products exceed the EFSA UL, which is 8.3-fold below the US UL, and about one in eleven exceeds the 50 mg/day that Australia will move behind the pharmacy counter. The Dutch experience suggests that a dose cap changes the reporting pattern for neuropathy [7]. EFSA took its reference point of 50 mg/day from the 1987 series [2], in which 48% of the women who had taken less than 50 mg/day for more than 6 months had neuropathy symptoms; an uncertainty factor of four gave 12.5 mg/day, a study in dogs gave 11.7 mg/day, and the midpoint, rounded down, became the UL of 12 mg/day [6]. The two limits differ in their starting evidence: the Institute of Medicine's 1998 value rests on small clinical studies of pyridoxine treatment at 100 to 300 mg/day [5], whereas EFSA's rests on that series, case reports and vigilance data at lower doses [6]. The earlier DSLD study of multivitamin products for children aged 1 to 3 found folic acid, vitamin A and zinc above the UL in a substantial share of products [12]; the present census adds vitamin B6, including doses printed for children 4 years and older that exceed the UL for that age by up to 6.2 times.

Regulatory and clinical implications

With potency limits barred by statute for supplements sold to adults in general [10], the US levers are label warnings, the UL itself and clinician awareness. Australia is moving its highest-dose products behind the pharmacy counter [8]; in the US, an advisory statement about peripheral neuropathy on labels at or above the UL would reach the 575 products identified here without restricting sale. The 1998 UL predates the vigilance data and most of the case reports that EFSA weighed, and it has not been reassessed. For clinicians, the practical message is that vitamin B6 doses of 100 mg/day or more are directed by products sold for sleep, mood, women's health, heart health and general supplementation, so a medication and supplement history for unexplained sensory neuropathy should include the vitamin B6 content of every multi-ingredient product. The children's findings describe printed directions rather than observed harm, but a label that gives one dose to "adults and children four years and older" at five or six times the children's UL is a labeling problem on its own terms, and the statutory bar on potency limits does not extend to products represented for use by children under 12 years [10].

Implications for research that uses DSLD

DSLD is a transcription, and this census found two error types that any dose study must handle. Twelve records stated an amount that contradicted the label's own per cent daily value, usually by a factor of ten. Another 21 records encoded the serving directions in a way that doubled or otherwise misstated the daily dose, most often by storing "two capsules twice daily" as a four-capsule serving taken twice; 17 of the 21 corrections lowered the dose. A study that multiplies DSLD's serving fields without reading the printed directions will therefore overstate maximum doses, and the products it names as extreme will include encoding errors. The parser rules described in the Materials and Methods, each forced by a real record, are published with the code so that they can be reused or challenged.

Limitations

This is a label census, not a sales census: a product sold by the million and a product sold by the dozen each count once, so the results describe the market offered to consumers, not population exposure; linking DSLD to national dietary survey data would be needed to estimate how many adults take 100 mg/day or more. DSLD's on-market flag can lag discontinuation, so some listed products may no longer be sold, and the database does not hold every product on the market. Labelled amounts are not measured content; manufacturers add overages, so actual content tends to exceed the label, and the labelled dose is more likely to understate than to overstate what a product contains. The UL is a population safety benchmark, not a toxicity threshold for an individual, and exceeding it is not an overdose or evidence of harm. The maximum directed dose overstates typical use; the single-serving and smallest-dose analyses bracket it. Product categories came from names and DSLD product types and are approximate. Pyridoxal 5'-phosphate and pyridoxine alpha-ketoglutarate labels state compound weights rather than vitamin B6 content; the main analysis takes the label as printed, and the conversion changed no count at the UL, although it roughly halves the highest doses. The search relies on DSLD's ingredient groups: a post-review probe found eight labels filed under a compound-specific group that the nine original spellings did not reach, and another group of that kind could exist. For 332 products, the twin labels disagreed on dose; the newest label was used, and counting any or every label moved the estimate by at most 16 products. The verification was done by one reviewer, with every decision published rather than independently repeated, and it lowered the estimate rather than raising it. The confidence intervals describe the precision of a snapshot taken as one state of a changing market; for the database itself the proportions are exact. Finally, the children's analysis depends on how a label words its directions and on DSLD's encoded serving record; a label whose encoding understated a printed child direction could escape the 30 mg/day screen, and products that print no age-specific dose but are plausibly used by children were not counted.

## Conclusions

In the US market, DSLD records as of October 4, 2026, indicate that a substantial proportion of adult vitamin B6 supplements recommend daily doses at or above the US tolerable upper intake level (UL), with some products exceeding this limit through the product alone and single-tablet doses reaching exceptionally high levels. Many high-dose products are marketed for purposes other than vitamin B6 supplementation. A considerable proportion also exceed the lower European UL, while some products recommend doses for children and adolescents that exceed the age-specific UL. The findings support a required neuropathy warning on high-dose labels, a reassessment of the 1998 UL in light of the evidence that other authorities have weighed, and routine attention to vitamin B6 content when neuropathy is evaluated. The deposited snapshot and code allow the census to be repeated as the market and the database change.
